# The impact of copayments on mental healthcare utilization: a natural experiment

**DOI:** 10.1007/s10198-017-0921-7

**Published:** 2017-08-03

**Authors:** Timo R. Lambregts, René C. J. A. van Vliet

**Affiliations:** Institute of Health Policy and Management, Erasmus University Rotterdam, Burgemeester Oudlaan 50, 3062 PA Rotterdam, The Netherlands

**Keywords:** Health insurance, Cost sharing, Copayments, Healthcare utilization, Mental healthcare, Natural experiment, D12, I13

## Abstract

Empirical evidence suggests that people are fairly sensitive to cost sharing arrangements in ambulatory mental healthcare. However, pure cost sharing effects are typically hard to measure due to the presence of adverse selection effects. In this paper, we examine the impact of cost sharing on mental healthcare utilization in the context of mandatory health insurance where adverse selection is absent. Using a large proprietary dataset of a Dutch private health insurer, we examine to what extent a new copayment scheme for adult mental healthcare changed healthcare utilization. We exploit the fact that non-adults are exempted from copayments. First, we compare changes in utilization among adults and non-adults using *t* tests and a difference-in-difference analysis. Second, we highlight differential changes in mental healthcare utilization by treatment (duration and type of mental illness) and individual characteristics (gender and socioeconomic status). Third, we evaluate to what extent anticipatory behavior occurred pending the introduction and subsequent repeal of the new copayment scheme. Our results show a strong and significant (*p* < 0.01) decrease in outpatient secondary mental healthcare utilization among adults following the introduction of copayments, which is absent among non-adults. This decrease is concentrated among treatments for less severe mental illnesses. Furthermore, the utilization patterns suggest the presence of anticipatory behavior.

## Introduction

The effects of cost sharing seem particularly strong for mental healthcare. Specifically, Frank and McGuire [1] show that ‘nearly all the available evidence, experimental or observational, points in the direction of greater price response for ambulatory [outpatient] mental health than other healthcare services’ (p. 911). Yet, with the exception of the RAND Health Insurance Experiment (HIE) [2], such observational research is subject to adverse selection [3]. Adverse selection likely leads to an underestimation of price responses for the population at large; when individuals can freely decide on the level of insurance coverage, healthy people are more likely to choose higher levels of cost sharing for which their response may be relatively small. Furthermore, evidence of price responses for mental healthcare outside the US is still mostly indirect and mainly comprises research on under- and overtreatment of mental disorders, rather than on price responses per se [4]. In contrast, this paper aims to investigate the pure copayment effects (i.e., without adverse selection effects) for outpatient mental healthcare in a non-US context where adverse selection does not play a role due to both mandatory health insurance and mandatory copayments. This is especially relevant as the benefits of copayments within Europe are increasingly being questioned by both scholars (e.g., [5]) and policy-makers [6].

In addition, this paper contributes to existing knowledge in two other ways. First, by estimating the differential impact of price on mental healthcare utilization by type of treatment, by gender, and by socioeconomic status, we contribute to the limited knowledge in this area. This is relevant because other studies indicate that particularly males and people with lower socioeconomic status are vulnerable to underutilization of mental health services [7]. Second, we examine whether people anticipate changes in copayments. Evidence on anticipatory behavior is limited, because most research is either survey-based [8–10] or prone to adverse selection effects [3]. Moreover, a recent empirical study shows that anticipatory behavior is important for an appropriate evaluation of the effect of cost sharing [11].

This research utilizes changes of copayments in the Dutch universal mandatory health insurance scheme to analyze price responsiveness for mental healthcare. In 2012, existing copayments for primary mental healthcare were raised and new copayments were introduced for secondary mental healthcare in the Netherlands. Using a large proprietary dataset of a private Dutch health insurer, we are able to examine the pure effect of these changes for outpatient mental healthcare. We do so by comparing changes in healthcare utilization between those who are affected by these changes in copayments (adults) and those who are not affected (non-adults).

In sum, the goal of this paper is threefold and consists of: (1) estimating the pure demand response for outpatient mental health services, net of selection effects and in another setting than the US; (2) estimating differences in demand varying with treatment, gender, and socioeconomic status; and (3) evaluating the occurrence of anticipatory behavior in response to changes in cost sharing regime.

### Previous research

Economic theory predicts that people use fewer mental healthcare services when cost sharing is introduced or increased in their insurance coverage. The magnitude of decreases in healthcare utilization depends on the extent of cost sharing and the elasticity of demand. The RAND HIE found a price elasticity of general healthcare between –0.10 and –0.14 for coinsurance rates between 0–25 and 25–95%, respectively [12]. Other research reported similar results in various countries and at various points in time [1, 13]. Research focusing specifically on outpatient mental healthcare suggests that the price elasticity of such care is larger than that of general medical care, as scholars found price elasticities of −0.79 and −0.31, respectively, for coinsurance rates between 25 and 95% [2]. Research in the Netherlands delivered similar results with price elasticities of −0.14 for cost sharing arrangements in general healthcare [14]. These elasticities differed greatly between healthcare services, with a price elasticity of −0.40 for visits to the general practitioner and −0.08 for prescription drug and were found to increase with the extent of cost sharing. Otherwise, most evidence of price effects in mental healthcare outside the US is still indirect. Notably, such evidence suggests that receiving a treatment is strongly associated with disorder severity as well as positively correlated with age, level of education, and the female gender [7].

There are three possible explanations for differences in price elasticities between mental healthcare and other healthcare services. First, it is argued that elasticities differ because of the necessity of treatments [14, 15]; it is presumably easier to forego a visit to a general practitioner for a minor ache than to forego a visit to the hospital for a broken leg. In the same way, a mental illness could be perceived as less acute than that same broken leg and could hence be easier foregone. Second, the willingness to seek professional help in mental healthcare is likely restrained by fears of stigmatization [7]. Third, an increasing number of people have pessimistic perceptions of the effectiveness of mental healthcare and sometimes even prefer to wait until a mental illness fades by itself [16]. Copayments could interact with and aggravate these tendencies to undertreat mental disorders and thus lead to differences in copayment effects vis-à-vis other healthcare services.

Furthermore, anticipation effects (or *ex ante moral hazard*) play a role in shaping responses to cost sharing. Price responses do not merely embody a binary choice between using and not using healthcare at a given cost sharing level. Rather, by adequately timing healthcare consumption such that healthcare is used when copayments are lowest, patients can minimize cost sharing. Changes in insurance coverage that are announced beforehand thus create opportunities for ex ante moral hazard if healthcare consumption can be scheduled. A recent study among employees whose firm discontinued a health plan with generous first-dollar coverage to only retain a high-deductible health plan for example found that this shift reduced healthcare utilization by 19% [11]. Yet, when correcting for anticipatory behavior, only an 11–15% decrease in healthcare utilization could be attributed to the high-deductible health plan. Hence, ex ante moral hazard may increase measured price elasticities in natural experiments by spurring demand prior to the introduction of new cost sharing arrangements to substitute for expected demand after that introduction.

### Empirical setting

The Dutch healthcare system is characterized by a universal mandatory basic health insurance scheme, covering all essential healthcare services with a standardized benefits package for the entire population. Basic health insurance coverage is offered by private health insurers in return for a community-rated premium. The basic benefits package, a mandatory deductible for most healthcare services and copayments, are all set by the national government.

The provision of Dutch mental healthcare can be distinguished in primary and secondary care. In our study period, 28% of the mental health patients received primary care and 77% secondary care [17]. Primary care, which is accessible without referral, offers treatments for relatively mild disorders. Secondary care consists of treatments of more serious conditions that need specialized care. In secondary mental healthcare, a further distinction can be made between curative care and long-term—often institutionalized—care. To gain access to secondary mental healthcare, a referral from a general practitioner or primary mental healthcare provider is required.

Since 2008, most mental healthcare services have been included in the basic health insurance, with the exception of chronic mental disorders and long-term mental healthcare,^1^ which are insured through a social long-term care insurance. Coverage for primary mental healthcare had been limited to eight sessions per year, all subject to a copayment of €10 per session. The cost sharing reforms, summarized in Table 1, encompassed both an increase of existing primary mental healthcare copayments and the introduction of a new copayment for secondary care. In primary care, existing copayments were increased from €10 to €20 per session and the number of sessions covered in the basic health insurance was reduced from eight to five. In secondary care, a copayment of €100 per 100 min, capped at €200 annually was introduced.^2^ These secondary care copayments were repealed again at the start of 2013. Furthermore, the reforms comprised the removal of adjustment disorders from the basic health insurance benefit package. At the same time, the mandatory deductible increased by €180 between 2011 and 2013. Finally, non-adults, constituting 23% of all Dutch mental health patients between 2011 and 2013 [17], were exempted from paying any copayments or deductibles between 2011 and 2013. This exemption hence creates a convenient control group to analyze the effects of introducing and increasing copayments.

**Table 1:** Cost sharing for adult mental healthcare between 2011 and 2013

Cost sharing	2011	2012	2013
Primary mental healthcare copayments	€10^a^	€20^b^	€20^b^
Secondary mental healthcare copayments	€0	€100/€200^c^	€0

^a^With a maximum of 8 sessions covered annually

^b^With a maximum of 5 sessions covered annually

^c^€100 per 100 min of treatment capped at €200 annually

### Data

This study utilizes proprietary anonymized claims data from a sample of individuals with a basic health insurance from a Dutch health insurer to analyze the number of mental healthcare treatments. Individuals in our sample that were not insured with this insurer for the entire period between 1 January 2011 and 31 December 2013 have been excluded in order to form an unvarying cohort. Individuals that made use of crisis treatments have been excluded from this sample as well, because such treatments were excluded from copayments.

In this way, we created a cohort of 324,675 continuously enrolled individuals. Of these, 78% were adults (≥18 years), 18% non-adults and 4% turned 18 during the period examined. This latter group has been excluded from further analysis, since, by turning 18, its individuals shifted from the control group to the treatment group during the period analyzed. The adult group consisted of 46% male and 54% female and for non-adults there was a 50/50 division. Subsequently, we estimated aggregated socioeconomic status scores (SES scores)^3^ of all individuals by linking their four digit zip codes^4^ to SES score data of The Netherlands Institute for Social Research [18]. Hence, we found average SES scores slightly below the national average of 2012: −0.12 for non-adults and −0.11 for adults. The aggregated SES scores were then used to assign the insured in our sample to a quintile, based on SES scores in the entire Dutch population. The distribution of individuals from our sample across these SES quintiles is summarized in Table 7 (see Appendix). Finally, we verified that changes in numbers of primary and secondary mental healthcare visits within our sample are comparable to national trends [17], signifying the external validity of our study.

To analyze healthcare utilization, we used so-called ‘diagnosis and treatment combination codes’ (DTC codes)^5^ and general billing information. Dutch health insurers register healthcare utilization of their insured through billing information from healthcare providers. In these bills, healthcare providers summarize treatments using DTC codes.^6^ For secondary mental healthcare, DTC codes include inter alia start and end dates of treatments, the illness that patients suffered (divided in 15 general diagnosis codes based on DSM-IV) and the total duration of the diagnosis and treatment (in ranges of minutes).^7^ For primary mental healthcare, no DTC codes exist and billing information only provides health insurers with dates of treatment sessions.

We utilize this data to determine when patients started their mental healthcare treatment, or *initial treatments*. For outpatient secondary mental healthcare, initial treatments exclude DTC codes that signify an extension of the treatment after 365 days. All other secondary treatments are considered initial treatments on the billed starting date. As primary care sessions are billed independently and without further detail, it is often unclear whether a consultation is a follow-up or signifies the start of a new treatment. Considering that on an annual basis five primary care visits are covered by the basic health insurance (one every 2.4 months), we assume primary mental healthcare sessions to be initial treatments when taking place three or more months after a previous primary care session. These initial treatments are measured per 10,000 insured per month. The number of initial treatments thus found, for both types of mental healthcare, are roughly normally distributed within years in our sample among both adults and non-adults.

## Methods

To evaluate the impact of copayments on mental healthcare utilization, we analyze changes of the monthly number of initial mental health treatments in our sample for both adults who faced changes in copayments and non-adults who did not face such changes. All analyses are performed using IBM Statistical Package for the Social Sciences (SPSS) version 23.0 for Windows. First, we perform paired *t* tests for the number of monthly initial treatments between the years 2011 and 2012 and 2012 and 2013 independently for both initial primary and secondary mental healthcare among non-adults and among adults. In addition, homogeneity of variance is tested by performing a Levene’s test alongside all *t* tests. These are followed by a difference-in-difference analysis between adults (treatment group) and non-adults (control group) over these two periods of time, using ordinary least squares (OLS) regression. We do so according to the following equation:

1 $$Y_{A,T} = \alpha + \beta_{A} \cdot A + \beta_{T} \cdot T + \beta_{A \cdot T} \cdot \left( {A \cdot T} \right) + \epsilon_{A,T}$$

This equation describes mental healthcare utilization (in average number of monthly numbers of initial treatments) (*Y*) as a function of adulthood (*A*) (minor = 0, adult = 1), time (*T*) (2011 = 0, 2012 = 1 or 2012 = 0, 2013 = 1) and time-differential adulthood effects, with error term $\epsilon$ and subject to parameters *α* and *β*. Subsequently, we analyze changes in secondary care utilization by separating secondary mental healthcare by kind of disorder treated and by duration of the treatment.

We expect to find significant changes in utilization for adults in secondary mental healthcare, while such changes are expected to be absent among non-adults. Although non-adults and adults are not completely similar groups, there is no reason to believe their mental healthcare utilization trends are not similar ceteris paribus. The hypothesized differential utilization trend would hence be attributable to the introduction of copayments for adults only. We also expect some impact of the copayments for primary care. Possibly, these changes are smaller than in secondary mental healthcare as the increase of copayments in primary care is smaller. On the other hand, illnesses treated in primary care are less serious than those treated in secondary care and are thus presumably easier to forego.

Second, we zoom in further on these effects by comparing the number of monthly initial mental healthcare treatments with the annual mean. Subsequently, we compare this with the annual standard error in order to analyze anticipation effects. Lack of data from earlier years, as well as converse effects of the introduction and repeal of copayments prevent a more sophisticated analysis, correcting for seasonality and annual trends. As anticipatory behavior presupposes awareness of the policy changes among the population, we have also tried to evaluate levels of awareness. Figure 2 (see Appendix) gives an overview of the utilization of related search terms in Google and links this to events surrounding the development of the new deductible policy and its repeal.

Third, we analyze to what extent differential effects of copayments exist between men and women and between different SES quintiles. To do so, we estimate the following equations:

2 $$Y_{G,T} = \alpha + \beta_{G} \cdot G + \beta_{T} \cdot T + \beta_{G \cdot T} \cdot \left( {G \cdot T} \right) + \epsilon_{G,T}$$

3 $$Y_{SES,T} = \alpha + \beta_{SES} \cdot SES + \beta_{T} \cdot T + \beta_{SES \cdot T} \cdot \left( {SES \cdot T} \right) + \epsilon_{SES,T}.$$

These equations describe *Y* in a similar way as Eq. (1) and as a function of: (2) gender (*G*) (male = 0, female = 1), time (T) and time-differential gender effects; and: (3) as a function of SES quintile (SES quintile *A* = 0, SES quintile *B* = 1), time, and time-differential SES quintile effects, respectively. We employ an OLS regression accordingly to estimate regression coefficients between men and women as well as regression coefficient between all pairs of SES quintiles.

## Results

Paired *t* tests show that the monthly number of initial secondary treatments for adults differs significantly between consecutive years in the period 2011–2013. Results of these tests are summarized in Table 2. In 2012, the number of initial secondary treatments per 10,000 insured dropped with 11.72 initial treatments (35%), compared to 2011 (*p* < 0.01). As hypothesized, no significant changes are found for mental healthcare utilization among non-adults. Neither are significant changes in initial primary treatments utilization detected among adults; *t* tests show only small and non-significant decreases in initial primary visits between 2011–2012 and 2012–2013. These results are robust and hold when the number of initial treatments is measured per week or per 2 weeks instead of per month. The variation in monthly number of initial treatments moreover satisfies homoscedasticity.^8^ A difference-in-difference analysis of the outpatient secondary mental healthcare utilization of adults and non-adults over the same periods of time confirms these results. This analysis reveals a significant (*p* < 0.01) time-differential utilization change between adults and non-adults in 2012 as compared to 2011 (Table 3).

**Table 2:** Paired *t* tests for monthly initial mental healthcare treatments between consecutive years

Years by type of care		Adults			Non-adults		
		Mean dif.	*t* value	*p* value	Mean dif.	*t* value	*p* value
Primary care	2011–2012	−0.85	−0.95	0.36	0.11	0.48	0.64
	2012–2013	−1.06	−1.50	0.15	−0.21	−0.76	0.45
Secondary care	2011–2012	−11.72	−9.65	0.00**	0.26	0.60	0.56
	2012–2013	1.44	1.47	0.14	−0.24	−0.49	0.63

* *p* < 0.05, ** *p* < 0.01

**Table 3:** Standardized coefficients for average number of monthly initial secondary mental healthcare treatments after OLS regression

Independent variable	2011–2012		2012–2013	
	*β*	*p* value	*β*	*p* value
Adulthood (*A*)	1.16	0.00**	0.98	0.00**
Time (*T*)	0.00	0.99	−0.08	0.86
$A \cdot T$	−0.36	0.00**	0.01	0.92

*p < 0.05, ** *p* < 0.01

Focusing on the significant decrease in secondary care utilization among adults in 2012, results display that utilization decreased across many of the existing 15 diagnosis codes. Table 4 shows *t* tests performed on the monthly number of initial treatments by diagnosis code between 2011 and 2012. The results demonstrate that the relatively strongest decreases in treatment utilization can be found among “vague” diagnosis codes: ‘unknown diagnoses’, ‘group rest diagnoses’^9^ and ‘other conditions that may be a cause for concern’. Additionally, the utilization of treatments for ‘adjustment disorders’ seems to have evaporated almost completely after the removal of these disorders from the basic health insurance benefits package.^10^ Moreover, treatments for ‘alcohol-related disorders’ also decreased significantly, highlighting the price responsiveness of these treatments.

**Table 4:** Paired *t* tests for monthly initial secondary mental healthcare treatments by diagnosis code between 2011 and 2012

Diagnosis code	Mean dif.	*t* value	*p* value
Unknown diagnoses	−8.79	−17.88	0.00**
Other disorders in childhood	0.02	1.50	0.13
Pervasive developmental disorders	−0.13	−1.28	0.22
Attention deficit disorders and behavioral disorders	−0.14	−1.63	0.12
Group rest diagnoses	−1.56	−15.21	0.00**
Adjustment disorders	−1.97	−6.28	0.00**^a^
Other conditions that may be a cause for concern	−1.39	−7.18	0.00**
Delirium, dementia and amnestic and other cognitive disorders	−0.05	−0.83	0.42
Alcohol-related disorders	−0.31	−4.94	0.00**
Other disorders related to an agent	−0.10	−1.19	0.24
Schizophrenia and other psychotic disorders	−0.10	−0.99	0.33
Depressive disorders	0.81	1.63	0.12
Bipolar and other mood disorders	0.05	0.90	0.38
Anxiety disorders	1.09	1.35	0.19
Personality disorders	0.26	0.86	0.40

* *p* < 0.05, ** *p* < 0.01

^a^A Levene’s test found heteroscedasticity of variation

Distinguishing by treatment duration, significant and substantial decreases are found for short treatment durations between 2011 and 2012 as well as significant increases in treatments of the shortest and the longest duration in 2013. These results are summarized in Table 5. Notably, when separated by duration, in 2013 we find significant increases in utilization of treatments of 0–250 min and ≥6000 min in duration, while in general there has been no significant increase in initial secondary treatments. Still, the increase in 2013 for initial secondary treatments of 0–250 min of 0.75 per 10,000 insured does not outweigh the 2012 decrease of 4.08 treatments. Finally, it is important to note that treatments of shorter duration are overrepresented among “vague” diagnosis codes. Hence, decreases in treatment utilization seem concentrated among treatments with “vague” diagnosis codes, treatments of short duration and among treatments that are both of short duration and with a “vague” diagnosis code.

**Table 5:** Paired *t* tests of monthly initial secondary mental healthcare treatments by duration

Treatment duration (min)	2011–2012			2012–2013		
	Mean dif.	*t* value	*p* value	Mean dif.	*t* value	*p* value
0–250	−4.08	−16.60	0.00**	0.75	3.47	0.00**
250–1800	−10.62	−9.84	0.00**	0.08	0.07	0.95
1800–6000	0.20	0.53	0.50	0.76	1.94	0.07
≥6000	0.04	0.70	0.49	0.18	2.29	0.03*

* *p* < 0.05, ** *p* < 0.01

**Fig. 1:**
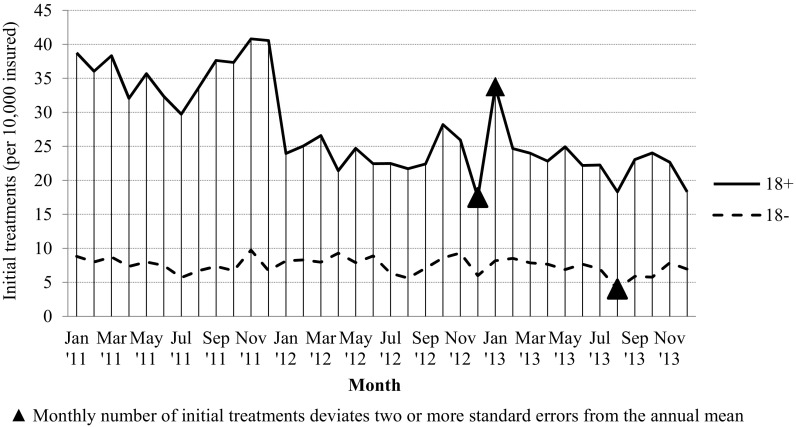
Monthly initial secondary mental healthcare treatments (per 10,000 insured)

**Fig. 2:**
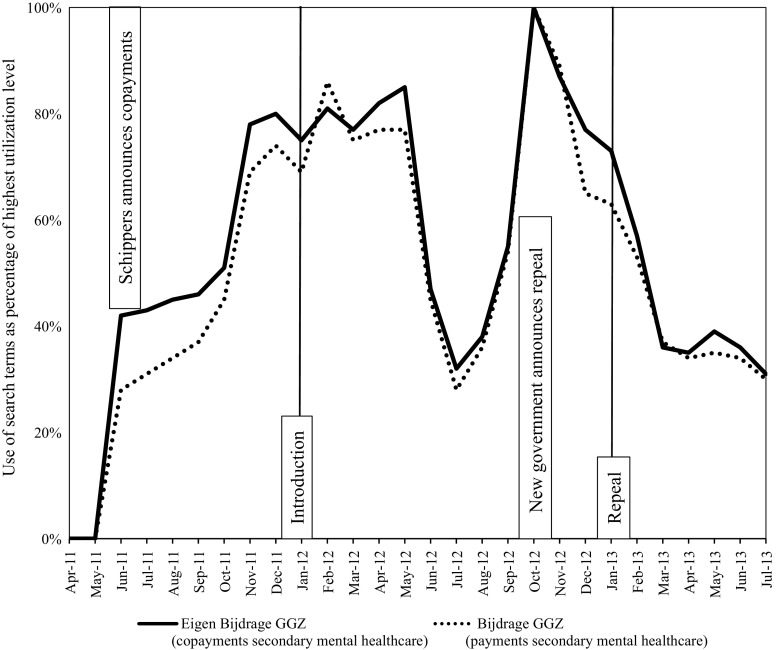
Use of copayment-related search terms in Google and matching events between April 2011 and June 2013. Data for search term utilization has been retrieved from Google Trends [23]

Concerning anticipatory behavior, Fig. 1 reveals significant deviations from the annual mean of monthly initial secondary treatments among adults at two points in time: in December 2012 and in January 2013. Among non-adults, the only significant deviation from an annual mean is found in August 2013. This deviation seems to signify an annually recurring decrease in utilization in July and August that is especially prevalent among non-adults: the summer break. In addition, we find a non-significant increase in initial secondary treatments after the announcement of copayments in June 2011 until the introduction of copayments in January 2012. This increase bears similarities with our proxy for awareness of the introduction of copayment as summarized in Fig. 2 (see Appendix). Splitting these results by treatment duration, we find that for treatments of 250–1800 min there was a significant negative deviation in December 2012, and a significant positive deviation in January 2013. For treatments of 1800–6000 min in duration, we find a significant positive deviation in January 2013 and a negative deviation in December 2013. No other significant deviations from the annual means have been discovered. Hence, anticipation effects appear to be concentrated among treatments of moderate duration.

As for time-differential gender effects, we find that women in general have higher levels of initial secondary treatments, but that these levels decreased significantly more than those of men from 2011 to 2012 (Table 6). Hence, the introduction of copayments has decreased mental healthcare utilization among men, but did so more strongly for women, nearly equalizing the level of treatment seeking in both groups. Thus, copayments did not aggravate existing treatment inequalities between men and women. Rather, such treatment inequalities seem to have diminished in 2012, as especially women, who had previously been more likely to seek treatment, showed a larger reduction in healthcare utilization (38 vs. 30%).

**Table 6:** Standardized coefficients for average number of monthly initial secondary mental healthcare treatments after OLS regression

Independent variable	2011–2012		2012–2013	
	*β*	*p* value	*β*	*p* value
Gender (*G*)	0.92	0.00**	0.63	0.00**
Time (*T*)	−0.37	0.00**	−0.21	0.84
$G \cdot T$	−0.40	0.00**	0.20	0.84

* *p* *<* 0.05, ** *p* < 0.01

Examining time-differential effects between pairs of SES quintiles, we find no indications of different changes of healthcare utilization between the SES quintiles. All SES quintiles show a mental healthcare utilization level of 37–44 initial treatments per 10,000 insured in 2011. In 2012, this dropped to 24–29 initial treatments, with decreases in healthcare utilization among different SES quintiles varying from 32% for the lowest quintile to 35–36% for all other quintiles. Similarly, the analyses do not reveal significant time-differential effects between pairs of SES quintiles. Possibly, these findings are impacted by the use of aggregated SES scores to estimate individual SES scores.

## Conclusions

In this study, we examined the effects of changes in cost sharing in both primary and secondary mental healthcare in the Netherlands. We capitalized on the exemption of non-adults from copayments to form a control group. We employed *t* tests and OLS regressions to evaluate utilization differences among different years, within subgroups, and between various treatments. This adds to the existing copayment literature by estimating demand response without selection effects and with a natural control group.

First, our results show that the introduction of a secondary mental healthcare copayment of €200 was followed by a 35% decrease in initial treatments among adults, without selection effects. A similar decrease was absent among non-adults. The impact of the copayments was strongest among treatments of short duration and treatments with “vague” diagnoses. This provides further evidence that the way in which copayments affect healthcare consumption depends partially on the necessity of care. However, we find no changes in primary healthcare utilization for milder care needs. Presumably, this is because primary mental healthcare copayments were already in place and were only increased with €10 per visit in 2012.

Second, our findings confirm the existence of anticipatory behavior; in line with earlier research the data showed increased mental healthcare utilization prior to the introduction of copayments in 2012 and significantly reduced initial treatments prior to the repeal of copayments in 2013. This implies that the demand response excluding anticipation effects is lower than 35%. The anticipation effects are concentrated among treatments of relatively short duration, suggesting that anticipatory behavior is strongest where general utilization effects are strongest and that both effects vary with the necessity of care.

Third, we find some evidence for a differential impact of copayments: mental healthcare utilization decreased significantly more among women (38%) than among men (30%). We find no significant differences in utilization changes between SES quintiles. Possibly, this is due to the use of aggregated SES scores based on zip code to estimate individual SES scores. Still, our findings show lower decreases in healthcare utilization among groups that have been identified as underutilizing mental healthcare by existing research. Mental healthcare utilization decreased significantly less among men than among women and less—albeit not significantly—among the lowest SES quintile compared to other SES quintiles.

It is important to be aware of the limitations of our study when interpreting the results. We used data from one single Dutch health insurer. Although utilization trends of its insured are in line with national trends, it is possible that this has influenced our results. Furthermore, a general assumption of studies relying on healthcare provider data is that providers register treatments accurately and in good faith. In addition, our analysis evaluates mental healthcare utilization trends by various partitions independently. As we have noted, some correlation exists between these variables and should be taken into account when interpreting our findings. Furthermore, we assumed that differences in mental healthcare utilizations between the different years analyzed are attributable to the introduction and repeal of copayments. Yet, the increases in the annual mandatory deductible may also have had a downward effect on the demand for mental healthcare by adults in 2012 and 2013. This implies that the impact of the new copayment scheme in 2012 has probably been overestimated. The higher deductible could also partially explain why mental healthcare utilization has not returned to its pre-2012 level after the repeal of copayments in 2013.

Our results have important implications for policy-makers both in the Netherlands and in other countries. We find that copayments for secondary mental health significant have a strong impact on mental healthcare utilization. The utilization effects, moreover, are unevenly distributed among the population, indicating that implementing copayments may change the distribution of mental health across a population. At the same time, the existence of anticipatory behavior shows that policy changes concerning health insurance coverage should be carefully implemented. Finally, this research has not focused specifically on evaluating costs and benefits of the implemented policy nor on its mental health effects or (potential) long-term effects, which hence remain fruitful areas for future research.

## Appendix

See Table 7 and Fig. 2.

**Table 7:** Overview of the distribution of the sample over population SES quintiles

SES quintiles (%)	SES range	Percentage of total sample (%)
0–20	−5.93 to −0.48	29.3
20–40	−0.49 to 0.11	23.7
40–60	0.12 to 0.52	13.1
60–80	0.53 to 0.97	14.5
80–100	0.98 to 2.93	19.4

## Footnotes

1 Long-term mental healthcare treatments include psychiatric institutionalizations of at least 1 year in duration (at least 3 years in duration as of January 1, 2015).

2 Exemptions were made for crisis treatments, treatments of involuntarily hospitalization and treatments started after so-called interference care, in which social workers try to persuade worrisome healthcare avoiders to obtain the healthcare services they need.

3 These aggregated neighborhood SES scores are based on four neighborhood characteristics: average income, percentage of inhabitants with a low income (less than €9250 annually converted to Dutch price levels of 2000), percentage of low-educated inhabitants [highest level of completed education is primary education, pre-vocational education (VMBO) or lower vocational education (MBO-1)] and percentage of inhabitants without a job. The SES scores reflect deviations from the national average over the years 1998–2014.

4 Dutch zip codes consist of four numbers and two letters (e.g., 1000 AA) in which the numbers indicate a neighborhood or village and the letters indicate one or sometimes multiple streets within this area.

5 For a more detailed overview of the system of DTC codes, see [19].

6 It is important to consider that relying on data provided by healthcare providers has two consequences. First, around 66% of all patients with a mental condition do not receive any treatment [20]. This group is not included in such data. Second, to an extent healthcare providers have opportunities for upcoding, hence the DTC codes can moderately deviate from the actual situation [21].

7 In the period studied, treatment duration was not reported directly. Ranges of total duration of the diagnosis and treatment could be inferred from the reported tariffs. For example, a fee of 3297 euros could be matched to a treatment duration between 1800 and 3000 min.

8 Levene’s tests [22] have been performed alongside all *t* tests and found no heteroscedasticity between any pair unless stated differently.

9 ‘Group rest diagnoses’ include DSM-IV diagnoses that have not been assigned separate diagnosis codes such as disorders of impulse control, dissociative disorders, and sexual and gender identity disorders.

10 Exclusion of ‘adjustment disorders’ from the overall analyses summarized in Tables 2 and 3 did not alter the interpretation of our findings.
